# Osteochondral regenerative engineering: challenges, state-of-the-art and translational perspectives

**DOI:** 10.1093/rb/rbac109

**Published:** 2022-12-26

**Authors:** Srimanta Barui, Debolina Ghosh, Cato T Laurencin

**Affiliations:** Connecticut Convergence Institute for Translation in Regenerative Engineering, University of Connecticut Health Center, Farmington, CT 06030, USA; Connecticut Convergence Institute for Translation in Regenerative Engineering, University of Connecticut Health Center, Farmington, CT 06030, USA; Connecticut Convergence Institute for Translation in Regenerative Engineering, University of Connecticut Health Center, Farmington, CT 06030, USA; Department of Orthopaedic Surgery, University of Connecticut Health Center, Farmington, CT 06030, USA; Department of Chemical and Biomolecular Engineering, University of Connecticut, Storrs, CT 06269, USA; Department of Biomedical Engineering, University of Connecticut, Storrs, CT 06269, USA; Department of Materials Science and Engineering, University of Connecticut, Storrs, CT 06269, USA

**Keywords:** osteochondral regeneration, additive manufacturing, gradient porosity, *in vivo*, commercialization

## Abstract

Despite quantum leaps, the biomimetic regeneration of cartilage and osteochondral regeneration remains a major challenge, owing to the complex and hierarchical nature of compositional, structural and functional properties. In this review, an account of the prevailing challenges in biomimicking the gradients in porous microstructure, cells and extracellular matrix (ECM) orientation is presented. Further, the spatial arrangement of the cues in inducing vascularization in the subchondral bone region while maintaining the avascular nature of the adjacent cartilage layer is highlighted. With rapid advancement in biomaterials science, biofabrication tools and strategies, the state-of-the-art in osteochondral regeneration since the last decade has expansively elaborated. This includes conventional and additive manufacturing of synthetic/natural/ECM-based biomaterials, tissue-specific/mesenchymal/progenitor cells, growth factors and/or signaling biomolecules. Beyond the laboratory-based research and development, the underlying challenges in translational research are also provided in a dedicated section. A new generation of biomaterial-based acellular scaffold systems with uncompromised biocompatibility and osteochondral regenerative capability is necessary to bridge the clinical demand and commercial supply. Encompassing the basic elements of osteochondral research, this review is believed to serve as a standalone guide for early career researchers, in expanding the research horizon to improve the quality of life of osteoarthritic patients affordably.

## Graphical abstract

**Figure:**
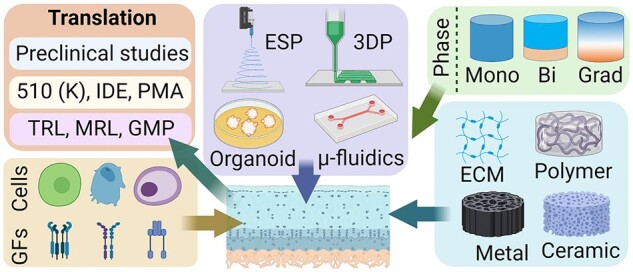

## Introduction

The osteochondral tissue is highly complex in terms of the spatial distribution of the structural elements, biochemical composition and their individual mechanical properties at each tissue level [1–3]. Broadly, Osteochondral tissue structure consists of articular cartilage, middle zone, deep zone, calcified zone and subchondral zone from distal to proximal direction. Osteoarthritis (OA) is one of the most prevalent forms of arthritis caused by osteochondral degeneration which affects 32.5 million people in the USA [4, 5] OA affects both the articulating cartilage and the subchondral bone, arising from a chronic partial or full thickness osteochondral lesion. A localized imbalance between anabolic and catabolic activities of the cartilage tissue is compromised leading to further advancement of the tissue degradation [6].

If detected early, common palliative measures for OA include physiotherapy coupled with non-steroidal anti-inflammatory drugs, intra-articular injection of glucocorticoids, hyaluronic acid (HA) etc., which do not have uniform effectiveness in the diverse patient population [7–9]. In a comparatively progressed stage, microfracture and micro-drilling are frequently used as clinical treatments. Unfortunately, viscoelastic hyaline cartilage is rarely observed in the regenerated tissue, whereas fibrocartilage comprising collagen type-I dominates the microstructure. As a result, articulating capability is often compromised leading to secondary osteochondral disease hotspots. Autografting of small sections of the osteochondral units from a non-articulating site is often transplanted in the affected area, a popular clinical procedure known as mosaicplasty [10, 11]. Unfortunately, allogenic osteochondral tissue transplantation is often associated with the risks of infection and disease transmission and inferior integration [12–14].

To address the above-described limitations, ‘Regenerative Engineering’, coined by Dr Cato T. Laurencin [15] is believed to be the next-generation approach in osteochondral regeneration. Regenerative engineering is defined as the convergence of the knowledge and concepts from disparate fields including advanced material science, stem cell science, physics, developmental biology and clinical translation for the regeneration of complex tissues and organ systems [16–20]. The last two decades have seen a wide range of regenerative approaches to develop osteochondral scaffolds employing novel biomaterials, defect-specific design concepts, tissue-specific stem cells, biomolecules and growth factors using conventional and advanced fabrication methodologies. The graphical abstract illustrates the current gamut of the highly inter-disciplined osteochondral research footprint. In this review, a comprehensive discussion of the key challenges, the existing state-of-the-art as well as the translational perspectives in osteochondral regeneration is presented.

## Current challenges in osteochondral defect regeneration

### Hierarchical complexity of osteochondral tissue structure and composition

**Figure 1.**
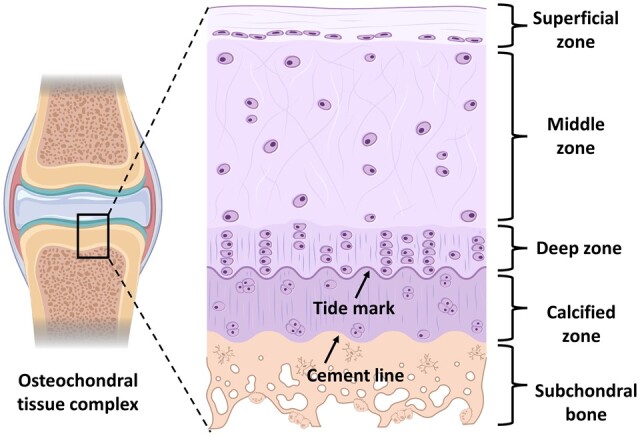
Gradient microstructure of a representative osteochondral tissue-complex cross-section (source courtesy with publication license: biorender.com).

Osteochondral tissue is a complex structure with multiple hierarchies (Fig. 1). The very first layer is the articular cartilage devoid of blood, lymphatic vessels or nerves, instead made up of a dense extracellular matrix (ECM) and sparsely embedded chondrocytes [21, 22]. The load-bearing articular cartilage (hyaline) consists of glycosaminoglycans (GAGs) including chondroitin sulfate (CS) and keratin sulfate, attached to the proteoglycans (mostly aggrecan) in the cartilage ECM. Being negatively charged, the GAGs attract water molecules assisted by hydrogen bonding and trap them inside the intermolecular spaces [23]. The abundance of the water molecules enables the articular cartilage in withstanding large compressive forces during the physiological loading cycles [24–26]. The viscoelastic nature of the hyaline cartilage is significantly contributed by the abundant collagen type II whereas collagen types V, VI, IX and XI are also present in the ECM microstructure [27–29].

The superficial layer has lesser thickness with a high density of collagen fibers oriented parallel to the surface with aligned flat chondrocytes. Being in contact with the synovial fluid, this zone maintains lubrication and wear resistance. Next to the superficial zone, is the middle zone consisting of columnar as well as oblique alignment of collagen fibers and sparsely distributed chondrocyte cells. The typical fiber orientation and the abundance of proteoglycan molecules ensure efficient shock absorbance and compressive stress resistance without eliciting any damage to the subchondral bone. Below the middle zone, the bunch of collagen fibers are perpendicularly oriented and anchored with the subchondral bone region, known as the deep zone. The oriented fibers contribute to the toughness of this area and the interstitial chondrocyte cells often align with the fibers in a grouped manner [30]. Calcified cartilage is the next tissue type below the deep zone, having a wave-like line distinguishing it from the deep zone, also known as the tidemark. Tidemark is believed to be a defensive front against the articulating shear forces on the columnar fibers anchored with the subchondral bone. In this zone, the chondrocytes also deposit collagen type X to calcify the ECM [31, 32].

Being separated from the calcified cartilaginous region by the cement line, the subchondral bone is the terminal layer, comprising trabeculae and bony lamella (subchondral bone plate) [23]. Being mechanically the strongest component in the osteochondral tissue complex, this zone plays a pivotal role in the force transmission through the joint. Reportedly, around 30% of the applied joint load is attenuated by the subchondral bone whereas the value for the same in the case of cartilage is nearly 1–3% [33, 34]. Furthermore, subchondral bone contains unmyelinated nerve endings, making it susceptible to nociception during cyclic loading on the eroded or damaged osteochondral unit. Thus, the regeneration of the seamless gradient between the innervated, vascularized and mineralized bone and the avascular, non‐mineralized and aneural cartilage should be one of the most challenging tasks to preserve the articular cartilage–subchondral bone homeostasis [35, 36].

### Challenges in cell sourcing and distribution

Autologous chondrocyte harvesting, isolation, expansion in culture, seeding in scaffolds or direct implantation face significant challenges due to limited density in the host cartilage tissue and their proneness of dedifferentiation [23, 37]. Thus, progenitor cells are considered as one of the popular choices in regenerative engineering. Human mesenchymal stem cells (hMSCs), bone marrow-derived stem cells (BMSCs) and adipose tissue-derived stem cells (ADSCs) are primarily used to allow tissue-specific differentiation in different zones (cartilaginous or bony). It is indeed a challenging exercise to spatially and temporally control the osteogenic and chondrogenic differentiation using a set of different matrix properties (biomaterials, scaffolds) and external factors (growth factors, small biomolecules, physical stimulation etc.). Although cell-free biomaterials-based scaffolds are commonly used commercially, it has been reported that cells, protein and growth factor-laden scaffolds exhibit better tissue regeneration *in vivo* [38–40]. It is worth mentioning, there are strict regulatory controls on cells and biological molecules laden osteochondral scaffolds in clinical practices. Besides the different cell types, there are also gradients in the cell population throughout the distal to the proximal length of a given osteochondral tissue complex. It is noteworthy that the cell density reduces from the superficial toward the middle and deep zones by 59% and 67%, respectively [41, 42]. While chondrocytes are the abundant cell types in the cartilaginous zones, the subchondral bony region comprises a spectrum of cell types such as osteoblasts, osteoclasts, osteocytes, chondrocytes, endothelial and MSCs where osteocytes comprise 90–95% population of the total bone cells regulating the interaction between the osteoblasts and the osteoclasts. It is, therefore, pertinent to understand the gradient of different cell types and their population density to better mimic the host tissue microstructure during the development of the matrix/scaffolds employing osteochondral regenerative engineering.

### Challenges in biomaterials, biomolecules and growth factors selection

When the repair/regeneration of the highly complex osteochondral tissue is concerned, the major challenge is to determine how perfectly the local tissue growth can be supported by either a biomaterial matrix or a scaffold, closely imitating the host tissue environment. Given the diverse material composition of the indigenous osteochondral tissue, it is essential to select suitable biomaterials for each layer, while designing the gradient or multi-layered (stratified) tissue constructs. There exists four major class of biomaterials contributing to osteochondral regeneration: natural polymers, synthetic polymers and inorganic biomaterials such as bioceramics and metallic biomaterials. *Natural polymers:* cellulose, silk, polyester, polyamides, collagen, alginate, chitosan (CS), gelatin etc. are the naturally derived polymeric biomaterials used for the regeneration of the cartilage zones. Very often, they are combined with bioceramics (mostly calcium phosphates) to provide higher mechanical stiffness to biomimic the subchondral bone phase [43–47]. The major pros of natural polymers are the better biocompatibility and enhanced immune system acceptance, whereas the variability in the molecular weight, chemical composition in different samples and batches as well as inferior mechanical strength are the notable cons. *Synthetic polymers:* the synthetic polymers used in osteochondral regeneration, such as polylactic acid (PLA), poly(d,l-lactic acid) (PDLA), polyethylene glycol (PEG), polyglycolic acid (PGA), polyvinyl alcohol (PVA), polypropylene fumarate (PPF), polycaprolactone (PCL), polylactic-co-glycolic acid (PLGA), polyethylene (PE), ultra-high molecular weight PE (UHMWPE) etc., are reliable in terms of mechanical strength fidelity and compositional consistencies but at the cost of hydrophilicity, biocompatibility and biodegradability [48, 49]. Biodegradability is a major issue when osteochondral tissue engineering is concerned, where most of the synthetic biomaterials perform unsatisfactorily. Biopolymer: in recent years, new biopolymers are evolving with enhanced biodegradability, one of which is citrate-based biopolymers. Polyoctamethylene citrate is one such example demonstrating accelerated osteochondral regeneration with commendable biodegradability [50–52]. Over the last two decades, Dr Laurencin’s research group contributed significantly to polyphosphazene-based regenerative engineering to have a tuneable control over tissue regeneration and scaffold biodegradability [53–56]. The biodegradability is governed by the tailored incorporation of hydrolytically active side groups such as, imidazole, lactate, glycolate or with amino acid ester group functionalization. It is speculated that polyphosphazenes have great potential in osteochondral regenerative engineering [57]. *Metal and bioceramics:* for the osseous phase, mostly calcium phosphates are used as the inorganic biomaterial whereas metallic biomaterials (titanium, Ti–6Al–4V, Co–Cr etc.) trabecular architectures are also reported but to a comparatively lesser extent. Hydroxyapatite (HAp) is the major workhorse to model the subchondral bone alone or in combination with other polymeric biomaterials [58]. Apart from HAp, tricalcium phosphate (TCP), tetracalcium phosphate, biphasic calcium phosphate, Bioglass^®^ etc. are also utilized to fabricate the subchondral bone phase of an osteochondral scaffold [59, 60].

Unlike the cartilaginous zone, the bony region is characterized by vasculatures and interconnected porosities. Very often, porogen materials, e.g. sodium chloride (NaCl), PEG, gelatin etc. are used as the sacrificial/leaching material to accord interconnected porosities in the micro-structure [61–64]. While it is rare to regenerate all the four intermediate zones of the cartilage individually, there is a common trend to imitate the cartilage and the bone as two different phases in bi-layered scaffolds, whereas three phases in the tri-layered scaffolds are considered such as, superficial (mostly hydrogel), intermediate (hydrogel and inorganic biomaterial) and the bony phases (thermoplastic biopolymers or bioceramics) [65, 66]. No distinguishable zonal demarcation should be noted in the gradient scaffolds where pure articular cartilage and bone mimicking biomaterials should be prevalent at the two extremities of the gradient scaffolds [23, 67, 68].

Growth factors and small molecules/drugs are of pivotal importance to strategically direct the chondrogenic and osteogenic differentiation of the stem/progenitor cells in the bioengineered scaffold or treatment/intervention region under interest. In the last two decades, it has been widely reported that TGFβ1, TGFβ2 and TGFβ3 promote cartilage regeneration whereas, BMP-2 and BMP-7 facilitate osteogenesis [69–73]. Insulin-like growth factors (IGF-1), recombinant human bone morphogenic protein (rhBMP-2) and recombinant human insulin-like growth factors (rhIGF-1) are also examples of highly cited growth factors to accelerate osteochondral regeneration. To induce vascularization in the subchondral bony region, researchers also made use of vascular endothelial growth factors, platelet-derived growth factors (PDGF), fibroblasts growth factors etc. Apart from the growth factor proteins, a set of biomolecules also demonstrated efficacy in osteochondral defect regeneration. While HA is a conventional choice to induce and direct chondrogenesis in tissue-engineered constructs or through direct intraarticular injection in defect sites, different peptides are also recently used to functionalize scaffold biopolymers to augment osteochondral regeneration. One such example is HA-binding (HAbind) PCL conjugate containing the amino acid sequence as found in the HA-binding region of the anchoring protein in the aggrecan complex, the key component of hyaline cartilage. Three glutamic acid peptides, known to differentiate hMSCs toward osteogenic lineage were used to modify the PCL (E3–PCL) [74]. The peptide molecules functionalized scaffolds demonstrated efficacious chondrogenesis and osteogenesis excluding the application of any growth factors. In addition, it has been shown as a wise alternative to use spatial concentration gradient of the growth factors and small biomolecules while loading in the tissue-engineered scaffolds to control the sustained release both in the culture media, *in vitro* and *in vivo*. Most often, core–shell or layer-by-layer deposition of the biochemicals is used to avoid post-implantation ‘burst’ release. Recently, in the Laurencin group, a novel artificial stem cell mimicking system is developed (synthetic artificial stem cells) with the capability to deliver the target-specific secretomes to regulate chondrogenesis in the OA treatment [75]. Similarly, extracellular vesicles (EVs) released by mesenchymal stem/stromal cells, recently grabbed the attention in view of their regulatory capabilities in targeted bone and cartilage regeneration. EVs mediate cell signalling, influences major biological activities such as cell growth, migration and proliferation. Briefly, the bi-lipid layered EVs breakdown in the extracellular space and the cargo is instantly released to interact with the target cells. Over the last 5 years, EVs are being investigated for their therapeutic efficacies in osseous and chondral regeneration [76–78].

### Challenges in adapting the microstructural and mechanical property gradients

The microstructure and mechanical properties of bioengineered scaffolds are directly related to each other, where the spatial porosity distribution influences the microstructure of the osteochondral scaffolds. The porosity and mechanical properties have an inverse relationship which is important to achieve the benefits of biological fluid exchange, cell migration, proliferation, vascularization as well as physiologically relevant strength properties to withstand the articulating loading cycles. The influence of porosity on chondrogenesis and osteogenesis is well established [41, 79]. Before developing osteochondral tissue architectures, it is imperative to understand the porosity distribution in a typical osteochondral tissue microstructure. The mean pore size, pore volume fraction and the interconnectivity from the articulating surface toward the subchondral bone vary widely. The extent and alignment of the proteoglycans and the collagen fibers control the porosity of the articulating layer which is normally 60–85% porous having the mean pore size in the range of 2–6 nm in diameter [41]. Being open and interconnected, the porosities enable the exchange of biological liquid from the synovial side to the subchondral bone region. These interconnected pores also facilitate the migration of BMSCs to the articulating surface to aid in chondrogenesis. Toward the calcified cartilage, the porosity further reduces making the zone almost impermeable except for the exchange of nutrients, signaling molecules and limited migration of stem cells which are important for the remodeling of the underlying tissues.

Similar to all osseous structures, the subchondral bone is also divided into two gradient zones, such as cortical and cancellous/spongy bone. The cortical bone interfaces with the calcified cartilage while the cancellous bone interacts with the bone marrow. The cortical bone is compact and mechanically strongest, having a pore volume fraction in the range of 5–30% and a mean pore size of 0.1–250 µm. The porosity increases gradually toward the cancellous site, having the pore volume fraction ranging from 30% even up to 90% depending on the anatomical site and the age. The mean pore size can vary in a broad range from 5 to 2000 µm (1.5–2 mm). Apart from the less densely packed trabecula, these areas contain plenty of blood vessels and unmyelinated nerve fiber terminals. Mechanical properties (Young’s modulus, compression/tensile/flexural strengths) directly rely on the porosity content in the microstructure [80–84]. It is recommended to conceive the ‘processing–structure–property’ (P–S–P) linkage of a novel architecture to endorse its efficacy in real-life osteochondral defect regeneration [85, 86]. The processing pathways generate the microstructure, whether the microstructure governs the end-properties of the scaffold/implant. Although it is not straightforward to imitate all the individual mechanical properties of the subsequent zones of an osteochondral site, it has been recognized as a consensus to simulate the average mechanical properties of the cartilage phase and the subchondral bone phase in the tissue-engineered constructs. Being a viscoelastic material (due to the presence of proteoglycans and water molecules), the hyaline cartilage also expresses frequency-dependent storage and loss modulus. The subchondral successive layers of the cortical and the trabecular bones exhibit similar mechanical properties to other bones found in different anatomical locations. Similar to cartilage, due to the anisotropic orientation of the collagen fibers and the minerals, the mechanical properties of bone depend on the direction of the force application. For example, the elastic modulus, compressive and tensile strength of cortical bone in the transverse direction are 10.1 ± 2.4 GPa, 131 ± 20.7 and 53 ± 10.7 MPa, respectively, while the values of the same parameters in the longitudinal direction are 17.9 ± 3.9 GPa, 205 ± 17.3 and 135 ± 15.6 MPa, respectively [41, 87, 88]. In general, mechanical properties of cancellous bone are direction independent due to the high porosity and random orientation of the trabecula; the elastic modulus and compressive strength vary in the range of 1–900 and 1–10 MPa, respectively [89, 90].

The gamut of conventional and advanced fabrication/processing methodologies should be adapted to manufacture osteochondral tissue-engineered constructs for strategic regeneration/repair. The selection of the zone-specific biomaterials, cells, growth proteins along with tailoring the processing-induced microstructure to achieve the local and global mechanical properties of the developed scaffolds are the need of the hour. Directional freezing followed by lyophilization, solvent casting, gas foaming, melt molding, injection molding, compression molding, sacrificial material leaching, phase separation process and polymeric microspheres sintering are a few examples of conventional fabrication methodologies.

## Current state-of-the-art in osteochondral defect regeneration

In compliance with the definition of Regenerative Engineering, it is essential to provide the host-mimicking microenvironment to the embedded cells to regenerate/replace the implanted scaffold with the newly developed tissues. To achieve this, the smart deployment of suitable fabrication methodologies is required to process or deliver the biomaterials, cells and growth factors with very high precision and controllability. In the last two decades, a significant number of studies have been reported to regenerate/repair osteochondral defects using both conventional and advanced processing technologies.

### Injectable and microsphere-based biomaterials in cartilage and osteochondral regeneration

In the early stage of OA, intra-articular knee injection using HA, pain suppressing corticosteroids, local anesthetics, analgesics as well as relatively newer regenerative approaches, such as platelet-rich plasma and autologous MSC injections are considered as the ‘first-line’ treatment modules (minimally invasive). Microsphere-based drug delivery captured major attention both in laboratory-based *in vivo* experiments as well as in clinical practices [91–93]. In this approach, the drugs or stem cells are encapsulated in biodegradable polymeric/hydrogel-based hollow microspheres and injected intra-articularly. The biodegradability is tailored to achieve control over the sustained release of the molecules for enhanced and long-term regenerative results. For example, recently, a novel biomimetic injectable amniotic hydrogel (AM) encapsulated ADSCs was delivered intra-articularly in a collagenase-induced osteoarthritic rat model to prevent inflammation and cartilage degeneration [94]. The efficiency of the inflammation prevention and cartilage regeneration was evaluated by qualitatively assessing the gross appearance of the regenerated tissue, and joint swelling as well as quantitative probing of the serum cytokine profiling and histology. It was found that at each time point (14, 21 and 28 days) of the post-treatment, the joint swelling was significantly decreased (0.05 < *P*) in the AM encapsulated ADSCs treated groups compared to the control demonstrating its anti-inflammatory effects in the osteoarthritic joint. The AM-ADSCs system also showed a significant reduction in the level of intercellular adhesion molecule 1 (ICAM-1), leptin, selectin and monocyte chemoattractant protein-1 (MCP-1) expressions both at 21 and 28 days post-treatment, along with the significant increment in TIMP-1 compared to the control groups at the same experimental time-points [94]. From the gross morphology and the histological analysis, it was found that the AM-ADSC treated joints demonstrated a significant reduction in synovial inflammation, and lesions-free smooth cartilage surfaces endorsed by the strong Safranin O staining.

Xu *et al.* [95] developed an injectable supramolecular gelatin-based host–guest macromer (HGM) system to deliver encapsulated growth factor (TGF-β1), small molecule (kartogenin, KGN) and stem cells (hBMSCs, rMSCs), to study their sustained release to mediate osteochondral tissue regeneration. The complex molecular assembly was developed and injected into the drilled femoral groove of the rat knee joint. From the biocompatibility perspective of the HGM supramolecular system, a prior subcutaneous implantation study showed 95% viability of the encapsulated hBMSCs. From the immunohistochemical staining results, it was observed that the deposition of type II Collagen (Col II) and CS, which are believed to be the two key components of the cartilage matrix [96–98], is higher in the HGM hydrogels than in the GelMA hydrogels. GAG quantification and histological staining results demonstrated the KGN/TGF-β1 injected HGM hydrogels as superior to KGN/TGF-β1 hydrogel, while both of them demonstrated better cartilage regeneration efficacies compared to unmodified control, *in vivo* [95]. Thus, injectable biomaterials along with cells, proteins and growth factors have promising therapeutic potential in the articular cartilage and osteochondral regenerative engineering.

### Conventional dual and multi-layered scaffold systems

In an attempt to mimic the layered native osteochondral tissue complex, the field of regenerative engineering significantly strived to develop an expansive variety of stratified scaffold systems having diverse structural and compositional combinations. The basic variant of the stratified scaffolds is bi-layered scaffolds where the two components resemble the cartilage and the subchondral bony layers, respectively. Albeit, it seems to be less rigorous to fabricate the bi-layered scaffolds, the risk of layer delamination persists in case of inferior interfacial interaction/bonding. The probability of failure is higher if the simulated layers are fabricated individually and joined subsequently. This remains applicable for multi-layered constructs as well [99, 100]. Notwithstanding, a good practice is to allow a common phase/overlapping zone/interface to reinforce both the layers together and simultaneously during fabrication. In the following sub-sections, we will highlight the bi-phasic and multi-phasic scaffold systems to regenerate osteochondral defects with an emphasis on both *in vitro* and *in vivo* results.

#### Dual layered/bi-phasic osteochondral scaffold system

Liu *et al*. [101] developed a biomimetic biphasic osteochondral scaffold with HA-hydrogel mimicking the articular cartilage, further strengthened by host–guest supramolecular units for sustained release of kartogenin (KGN, chondrogenic promoter). Alendronate (ALN—inducing osteogenesis) laden HAp scaffold was fabricated as the bone mimicking zone and combined with the cartilage layer with a semi-immersion technique. Post-fabrication micro-computed tomography revealed efficient penetration and integration of the successive layers. In the KGN-laden cartilage phase, the expression levels of the chondrogenic marker genes (aggrecan, collagen II and proteoglycan 4 precursor) of the hydrogel encapsulated hBMSCs upregulated after 21 days of *in vitro* culture. Similarly, the expression levels of the osteogenic marker genes (alkaline phosphatase, RunX2 and collagen I) in the ALN-laden HAp phase were upregulated multifold compared to the ALN-free HAp phase [101]. Taken together, the hydrogel-ceramic bi-phasic scaffolds demonstrated superior chondrogenic and osteogenic properties *in vitro*, which was further validated *in vivo* by implanting subcutaneously in a rat model. rMSCs were loaded in the scaffolds and the explants were characterized after 2 months of the implantation. Conformed to the *in vitro* results, the expression levels of all the chondrogenic and osteogenic marker genes were significantly higher in the KGN–ALN–laden scaffolds compared to the drug-free scaffolds [101].

More recently, Cao *et al*. [102] put a step forward to prepare ECM-specific dual-phase osteochondral scaffolds. It is known that decellularized ECM-based scaffolds provide natural chondrogenic and osteogenic microenvironments for stem cells [102–104]. In this study, the individual ECMs were stacked on top of each other in a cylindrical mold, followed by lyophilizing to obtain the bi-phasic osteochondral scaffold. BMSCs were seeded in the bi-phasic scaffolds and a significantly higher expression of the chondrogenic (aggrecan, collagen II and SOX9) and osteogenic marker genes (collagen I, OCN, RUNX2 and a higher ALP deposition) were recorded in case of the ECM based scaffolds compared to the untreated control groups. The osteochondral tissue regeneration was investigated in the trochlear grooves of rabbit femurs by histological analysis and immunohistochemistry. It was found that the ECM-based scaffolds significantly outperformed the untreated controls in osteochondral defects regeneration [102]. In a consensus, the cartilage layer is commonly engineered using a hydrogel material considering the higher water molecules up-taking capabilities to enhance the articulation and wear resistance resembling the native proteoglycan-rich articular cartilage. There is a spectrum of biomaterials available to simulate the bone layer, ranging from bioceramics to biopolymers [105–107].

#### Multi-phasic osteochondral scaffold system

With the concurrent evolution of host tissue-mimicking novel biomaterials and rapid advancement in tissue engineering strategies, the next generation of osteochondral regenerative engineering is spearheading toward a more biomimetic and architecturally complex scaffold system. In addition to the base bi-layers of cartilage and bone, the importance to regenerate the calcified cartilage interface in osteochondral tissue engineering is recently realized [31, 108, 109]. This interface is believed to be a diffusion barrier minimizing the fluid flow between cartilage and the subchondral layer, thereby preventing the blood vessel invasion from the bone phase.

In an early study, a multiphasic stratified scaffold system was developed using PLGA-Bioglass^®^ (BG) composite microsphere for the bone region and agarose gel as the cartilage layer with different cell types confined in different zones [110]. Chondrocytes were laden both in the agarose phase (first phase) mimicking functionalities and mechanical properties of the cartilage layer as well as in the transition phase of agarose and PLGA-BG (second phase) to simulate the calcified cartilage layer. In the PLGA-BG composite bony layer, osteoblasts were seeded to engineer the subchondral bone (third phase). It was found that the relatively stiffer matrix of the interfacial region facilitated the chondrocytes to be mineralized and form the calcified region. During the *in vitro* co-culture, the individual phenotypes of both chondrocytes and osteoblasts were maintained, where chondrocytes produce proteoglycans and type II collagen while osteoblasts deposited type I collagen and maintained the ALP activity. Although the calcified interface generation was observed in the transition zone, chondrocyte hypertrophy in the particular region was not determined [110].

In the recent year, a multi-layered bionic scaffold consisting of porous HAp (bone layer), 20% silk fibroin (SF, intermediate layer) and 5% SF (cartilage layer) using a repeated freeze–thaw method was developed [111]. The scaffolds were modified using polydopamine (PDA) and the cartilage layer was loaded with PDGF. Synovial MSCs (SMSCs) were seeded in the scaffolds and the capability of osteochondral regeneration was assessed in the trochlear zone of the rabbit knee joint [111]. The *in vitro* PDGF release in the PDA-modified scaffolds increased gradually with time. In line with the MRI and gross morphological assessment, the histological staining (H&E, Safranin O/fast green) and immunohistochemistry (collagen I, collagen II, aggrecan) analysis also showcased significantly higher regeneration capability of the PDA-PDGF scaffolds, *in vivo* [111].

**Figure 2.**
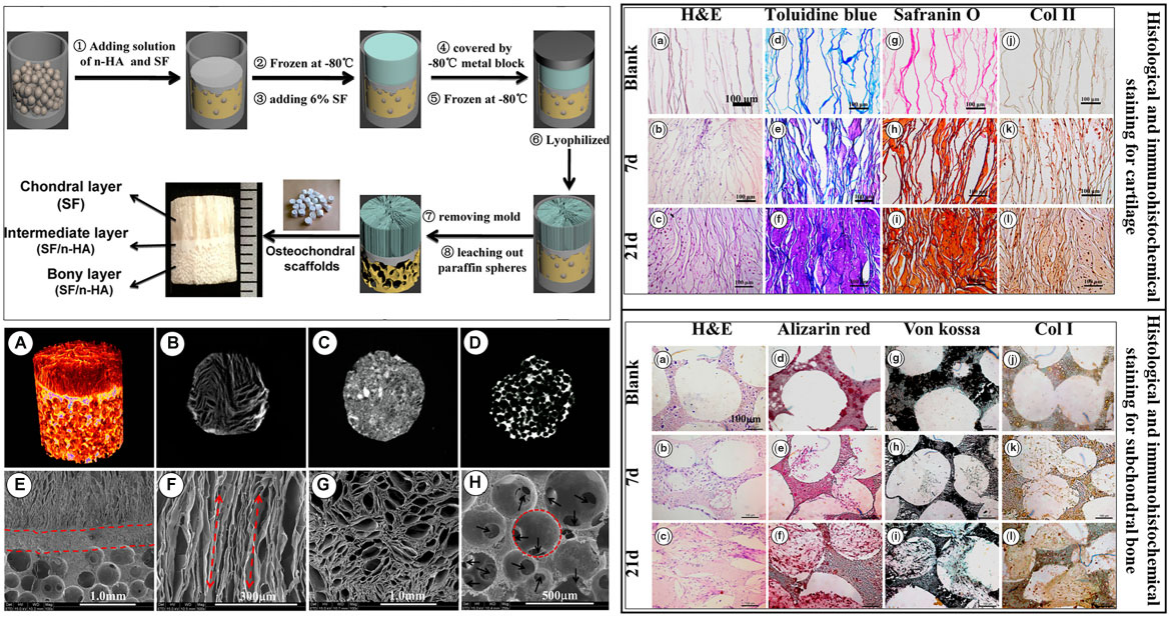
Left top panel schematically describes the tri-phasic scaffold manufacturing technology. Left bottom: (**a**–**d**) the micro-CT 3D volume rendered image. (**e**–**h**) SEM images of different region of the scaffolds. Red circle and the black arrows indicate macropores and the interconnection channels, respectively. Right top and bottom panels represent the histological and immunohistochemical staining images of cartilage and bone regions (reproduced with permission from American Chemical Society).

Ding *et al*. [112] employed a modified temperature gradient-guided thermal-induced phase separation (TIPS) technique to develop a unique osteochondral scaffold. The biomimetic chondral layer was composed of longitudinally oriented tubular SF and the bone layer was made up of spherical microporous HAp and SF composite [112]. In brief, HAp and SF composite solution was infiltrated in partially sintered paraffin microspheres in a mold and frozen followed by pouring of SF solution to allow rapid directional solidification. After lyophilization, the columnar SF phase was crystallized with methanol solution, and the partially sintered paraffin microspheres were leached out with hexane leaving interconnected spherical microporosities in the HAp-SF bone layer. The scaffolds were seeded with ADSCs tagged with phycoerythrin (PE)-conjugated anti-rabbit monoclonal antibodies (CD44-PE, CD105-PE and CD34-PE) to quantitatively probe the expressions in flow cytometry [112]. A cell-free compact region in between the cartilage and subchondral bone was observed closely resembling the intermediate calcified cartilage. In the chondral region, toluidine blue, Safranin O and immunohistochemical staining disclosed that GAG deposition and collagen II expression increased with incremental time. Similarly, in the bone region, alizarin red, von Kossa and immunohistochemical staining revealed higher matrix mineralization and collagen I expression over time. Figure 2 represents the scaffold preparation methodology and the histological and immunohistochemical results demonstrating the capability of the tri-layered bionic scaffolds in the regeneration of osteochondral defects [112].

#### Gradient osteochondral scaffold system

Gradient osteochondral scaffolds are the most reliable and consistent performers under physiological loading cycles due to the absence of an abrupt transition between dissimilar material properties/structural elements [1, 41, 68]. In normal native tissue also, a significant overlap and seamless transition between the adjacent zones of different tissue hierarchies are observed. There exists a broad gamut of technology varieties to develop gradient osteochondral tissue constructs encompassing both traditional and advanced methodologies. In this section, we will discuss the traditional methodologies while the advanced approaches will be discussed in upcoming sections.

Dorcemus *et al.* [68] developed a gradient PLGA–HAp–HA hydrogel-based scaffold system, where PLGA microspheres with a graded pore volume distribution were sintered to achieve gradient porosity distribution. In brief, the HAp-modified PLGA microspheres were mixed with varying quantities of NaCl porogen and stacked on top of each other followed by sintering. Next, the porogen was leached in water and the higher porosity end of the graded porous PLGA–HAp scaffolds was infiltrated with HA hydrogel to simulate the cartilage phase. Before infiltration, the PLGA–HAp scaffolds were dip-coated with BMP-2 growth factor and the hydrogel contained TGF-β1 along with hMSCs. Finally, the scaffolds were cultured in hMSCs laden co-differentiation media and the chondrogenic (DMMB assay) and osteogenic (alizarin red staining) potential were evaluated in the distal and proximal end of the gradient porous scaffolds, respectively [68]. Higher GAG deposition was observed in the distal cartilage end whereas there was no significant difference in the osteogenesis at the two ends of the scaffolds. In this study, the synergistic effect of BMP-2 and TGF-β1 in chondrogenesis was established where it was noted that Col II, Sox9, GAGs and aggrecan expressions increased with BMP-2 in a dose-dependent manner [68].

In another study, Mohan *et al*. [113] also fabricated PLGA-based scaffolds having opposing gradients of CS and β-TCP. Chondrogenic (PLGA–CS) and osteogenic (PLGA–TCP) monodispersed microspheres were separately prepared, and the microsphere suspensions were injected from mutually opposite directions in a cylindrical mold. The gradient chondrogenic and osteogenic scaffold was sintered at ambient temperature using a ‘solvent/non-solvent’ sintering technique using ethanol–acetone and lyophilized. The cylindrical scaffolds were infiltrated with either TGF-β3 or IGF-1 and implanted in the femoral condyles of skeletally mature sheep models [113]. After the single end-point at 52 weeks, the scaffolds were explanted and the tissue sections were stained using H&E and Safranin-O to probe the chondrogenesis. It was found that the regenerated hyaline cartilage in the animal group that received the scaffolds with TGF-β3, was significantly higher compared to the group who received IGF-1 modified PLGA–CS–TCP scaffolds. Surprisingly, no assessment for the osteogenesis was carried out in this study which may be due to regeneration of cartilage is more challenging compared to the bone counterpart [113]. In the authors’ group, microsphere-based biodegradable polymeric scaffolds are well established in bone regenerative engineering [114, 115].

In a recent study, a high throughput methodology was developed to create seamless gradient osteochondral scaffolds using a novel buoyancy-driven approach [116]. Polysaccharide Ficoll was added with GelMA (gelatin methacryloyl) as the denser base solution. Another solution of GelMA and HepMA (heparin methacryloyl) was added to the base solution with a controlled injection rate. The HepMA phase was pre-laden with BMP-2 and both the phases (base and injected) were pre-loaded with hMSCs and photoinitiator. After the formation of the stable buoyancy-driven gradient solution, the entire system was immobilized using UV light-mediated crosslinking [116]. The novel osteochondral construct was cultured in a common media for 28 days. Alizarin red S staining exhibited the formation of an osseous layer in the BMP-2 rich phase (HepMA side) while alcian blue revealed the sulfated GAGs throughout the length of the scaffolds. The same trend was endorsed by immunohistochemical analysis as well, where collagen type II was expressed throughout the scaffold and the bone marker protein osteopontin was found in the mineralized cap on the bony side. Table 1 summarizes the results of the studies discussed in this division.

**Table 1.** Current progress in the development of osteochondral tissue-engineered scaffolds using conventional manufacturing approaches

Scaffold categories		Composition	Brief fabrication strategy	Key results	Refs
Stratified	Bi-phasic	Bone layer: HAp Cartilage layer: HA hydrogel KGN and ALN loaded in cartilage and bone layers	Semi immersion technique. ALN-loaded HAp scaffolds were kept in a mold partially immersed in gelatin. In refrigerated conditions, the KGN-loaded pre-crosslinked HA hydrogel was poured on the top to infiltrate the leftover height of the HAp scaffold and fully UV crosslinked. Later the gelatin was removed from the bottom zone of the HAp scaffold in warm water.	1. Micro-computed tomography: efficient integration of the layers. hBMSc were cultured in the scaffolds	[101]
				2. In the KGN-HA phase, the expression of aggrecan, collagen II upregulated after 21 days of culture. Expression levels of ALP, RunX2 and collagen I in the ALN-HAp phase were also upregulated.	
				3. rMSCs were seeded in the scaffolds and implanted subcutaneously for 2 months. Expression levels of all the chondrogenic and osteogenic marker genes were significantly higher compared to the drug-free scaffolds.	
		Bone layer: bovine bone-derived ECM hydrogel Cartilage layer: bovine cartilage-derived ECM hydrogel	The individual ECMs were stacked on top of each other in a cylindrical mold. The ECM hydrogel complex was lyophilized together to obtain the bi-phasic osteochondral scaffold. Bone marrow-derived stem cells (BMSCs) were seeded in the bi-phasic scaffolds.	1. A significantly higher expression of the chondrogenic (aggrecan, collagen II and SOX9) and osteogenic marker genes (collagen I, OCN, RUNX2 and ALP) in the case of the ECM-based scaffolds compared to the untreated control groups	[102]
				2. *In vivo* histological staining (H&E, Toluidine blue, Safranin-O and fast green) and immunohistochemistry (collagen I and collagen II) showed that the ECM-based scaffolds surpassed the untreated controls	
				3. ICRS and O’Driscoll scores were consistently higher in the bi-phasic ECM-based scaffold compared to the untreated control group	
		Bone layer: SF and CaP composite Cartilage layer: SF	Salt leaching and freeze-drying. CaP nanoparticles by acid-base reaction in silk solution in presence of NaCl particles. NaCl particles are leached and fresh SF solution is added on the top of the microporous SF-CaP scaffold.	1. Enhanced attachment, viability and proliferation of RBMSCs *in vitro*	[107]
				2. Host tissue integration in rabbit critical OC defect with a layer of connective tissue adhered on the entire surface of the scaffolds, no signs of acute inflammation	
				3. The biphasic scaffolds showed cartilage regeneration in the silk layer, along with subchondral bone ingrowth and angiogenesis	
		Bone layer: PCL Cartilage layer: methacrylated HA	Salt leaching, infiltration and photocrosslinking. PCL was dissolved in chloroform with NaCl particles. The solvent evaporated, NaCl leached and the porous PCL scaffold was suspended in LAP-modified MeHA solution with bovine MSCs. Finally, the gel is UV photo-crosslinked	1. The mechanical properties of the MSCs encapsulated HA hydrogel/PCL osteochondral scaffolds matured over time, and obtained the peak compressive strength at a time when hydrogel/PCL interfacial shear strength was still developing.	[117]
				2. In a TGF-β3 supplemented culture media, the GAG (Safranin O/Fast green and alcian blue) and collagen (picrosirius red) contents increased over time indicating the zone-specific differentiability of MSCs.	
	Multi-phasic	Bone layer: PLGA-BG Intermediate layer: agarose and PLGA-BG Cartilage layer: agarose gel	BG-PLGA microspheres were sintered and agarose gel was partially infiltrated to make the transition region while pure agarose gel was retained on the top as the cartilage phase. Chondrocytes were laden in agarose and the PLGA-BG phase was seeded by osteoblasts	1. During co-culture, both chondrocytes and osteoblasts maintained their phenotypes, where chondrocytes produced proteoglycans and type II collagen while the osteoblasts deposited type I collagen and maintained ALP activity.	[110]
				2. Calcified interface generation was observed in the transition zone	
				3. Increment in chondrocyte density resulted in elevated ECM deposition and higher mechanical properties	
		Bone layer: HAp Intermediate layer: 20% SF Cartilage layer: 5% SF	HAp-5% SF solution casted in mold, lyophilized and sintered for porous HAp. Next, 20% SF solution was added on top of the porous structure followed by freeze drying to obtain the calcified cartilage layer. Finally, 5% SF solution was added on top of it and lyophilized. PDA modification of scaffolds and the cartilage layer was laden with PDGF. SMSCs were seeded in the scaffolds.	1. Capability of osteochondral regeneration was in the trochlear zone of the rabbit knee joint	[111]
				2. The *in vitro* PDGF release in the PDA-modified scaffolds was sustained in nature	
				3. In agreement with MRI and gross morphological assessment, histological staining (H&E, Safranin O/fast green) and immunohistochemistry (col I, col II, aggrecan) analysis demonstrated significantly higher regeneration capability of the PDA-PDGF scaffolds, *in vivo* (trochlear groove of rabbit femur)	
		Bone layer: HAp-SF Intermediate layer: cell-free intermediate layer induced *in vitro* Cartilage layer: 6% SF	HAp and SF solution infiltrated in partially sintered paraffin microspheres in a mold and frozen. 6% SF solution was poured on top of it and directionally solidified. Next, the entire unit was lyophilized. Columnar SF phase is crystallized with methanol and partially sintered paraffin microspheres were leached out with hexane leaving interconnected microporosities in the bony layer.	1. TIPS induced bionic columnar cartilage zone seamlessly with a simulated bone layer	[112]
				2. A cell-free compact region in between the cartilage and subchondral bone was observed which closely resembles the intermediate calcified cartilage in normal osteochondral tissue	
				3. In the chondral region, toluidine blue, Safranin O and immunohistochemical staining revealed the continued GAG deposition and collagen II expressions over time	
				4. Alizarin red, von Kossa and immunohistochemical staining in the bony zone revealed a time-dependent increment in matrix mineralization and collagen I expression	
Gradient		PLGA-HAp microsphere-based gradient scaffold with HA hydrogel infiltrated in the higher porous distal (cartilage) end	HAp-modified PLGA microspheres with varying vol% of NaCl stacked in a mold followed by sintering. NaCl was leached in water and the higher porosity end (distal) was infiltrated with HA hydrogel. PLGA-HAp scaffolds were pre-modified with BMP-2 and the hydrogel contained TGF-β1 along with hMSCs. Scaffolds were cultured in hMSCs laden co-differentiation media	1. Higher GAG deposition was observed in the distal cartilage end whereas there was no significant difference in the osteogenesis at the two ends of the scaffolds	[68]
				2. Synergistic effect of BMP-2 and TGF-β1 in chondrogenesis was established	
				3. Col II, Sox9, GAGs and aggrecan expressions increased with BMP-2 in a dose-dependent manner	
		PLGA-based scaffolds having opposing gradients of chondroitin sulfate (CS) and β-TCP	PLGA-CS and PLGA-TCP monodispersed microspheres were separately suspended and injected from mutually opposite directions in a cylindrical mold. The suspension media was filtered out and the gradient chondrogenic and osteogenic scaffold was sintered at ambient temperature using the ‘solvent/non-solvent’ sintering technique (ethanol/acetone).	1. From H&E and Safranin-O staining it was found that the *in vivo* cartilage regeneration in the scaffolds with TGF-β3, was significantly higher compared to the group who received IGF-1 modified PLGA-CS-TCP scaffolds.	[113]
				2. First long-term study in literature for critical-sized gradient osteochondral scaffolds in a large animal model where ‘microfracture’ was used as the control treatment	
				3. Hyaline cartilage regeneration in unmodified and growth factor modified PLGA-CS-TCP scaffolds overwhelmed the microfracture treated groups	
		GelMA with density modifier Ficoll as the base layer and HepMA as injected phase for a buoyancy-driven gradient.	A lower density HepMA phase was injected in a higher density GelMA base layer and after the formation of the stable buoyancy-driven gradient solution, the entire system was immobilized using UV crosslinking. The HepMA phase was pre-laden with BMP-2 and both the phases were pre-loaded with hMSCs and photoinitiator.	1. Alizarin red S staining showed the formation of osseous cap formation in the BMP-2 rich phase (HepMA side) while alcian blue revealed the sulfated GAGs throughout the length of the scaffolds	[116]
				2. Similar trend in immunohistochemical analysis, collagen type II expression throughout the scaffold and osteopontin expressed in the mineralized end	
				3. Raman spectroscopic analysis manifested the fingerprints of hydroxyapatite (predominant) and β-TCP in the mineralized layer	

### Advanced manufacturing strategies in developing biomimetic osteochondral scaffolds

Although the traditional fabrication technologies were deployed in a handful of successful osteochondral regenerative research, with the rapid advancement of the manufacturing schemes, osteochondral regenerative engineering also benefited largely to develop more bionic scaffolds with tuneable structural and functional properties. This section will review the progress of a few broadly used advanced manufacturing methodologies in osteochondral regeneration, e.g. electrospinning, different additive manufacturing (AM) and their combinations, and organoid and microfluidics-based regeneration modeling.

#### Electrospinning-based manufacturing of osteochondral scaffolds

Electrospinning, formerly known as electrostatic spinning, was first studied by Zeleny in the year 1914 whereas Formhals filed the first patent on the electrospinning process in producing polymeric nanofibres in 1934 [118–121]. Although the basic concept was established long before (1902) when Cooley [122] and Morton [123] patented the methodology and tools for electrically dispersing fluids, it was Laurencin *et al.* [124, 125] who established this technique for the tissue engineering purpose in the year 2002. The detailed electrospinning technology and the process physics can be found elsewhere [118, 126, 127]. Electrospinning is known for its multimodal capability to generate uniaxial, co-axial or multiaxial polymeric nanofibers which are collected on substrates to have highly interconnected micro- to nanoporous scaffolds having a very high surface area, one of the major prerequisites for enhanced cell–material interaction. Owing to the provision of multicore production, electrospun fibers are an excellent medium for target-specific controlled drug delivery [128].

Hejazi *et al*. [129], in a very recent study, prepared a five-layered osteochondral scaffold using a specially designed electrospinning set up. Bone and cartilage phases at the two extreme ends were deposited using PCL/gelatin + nano-HAp (nHA) and PCL/gelatin + CS/PVA-based nanofibers, respectively. In between these two layers, three intermediate layers were having continuously varying compositions of PCL, gelatin, CS and PVA to mimic the continuous compositional and mechanical gradient from bone toward the cartilage. Due to the interconnected porosity distribution and presence of gelatin, water uptake was boosted, facilitating the body fluids and nutrients transport from the host tissues. The Hap-based mineral nanoparticles enhanced the mechanical properties of the multi-layered electrospun scaffolds [129].

**Figure 3.**
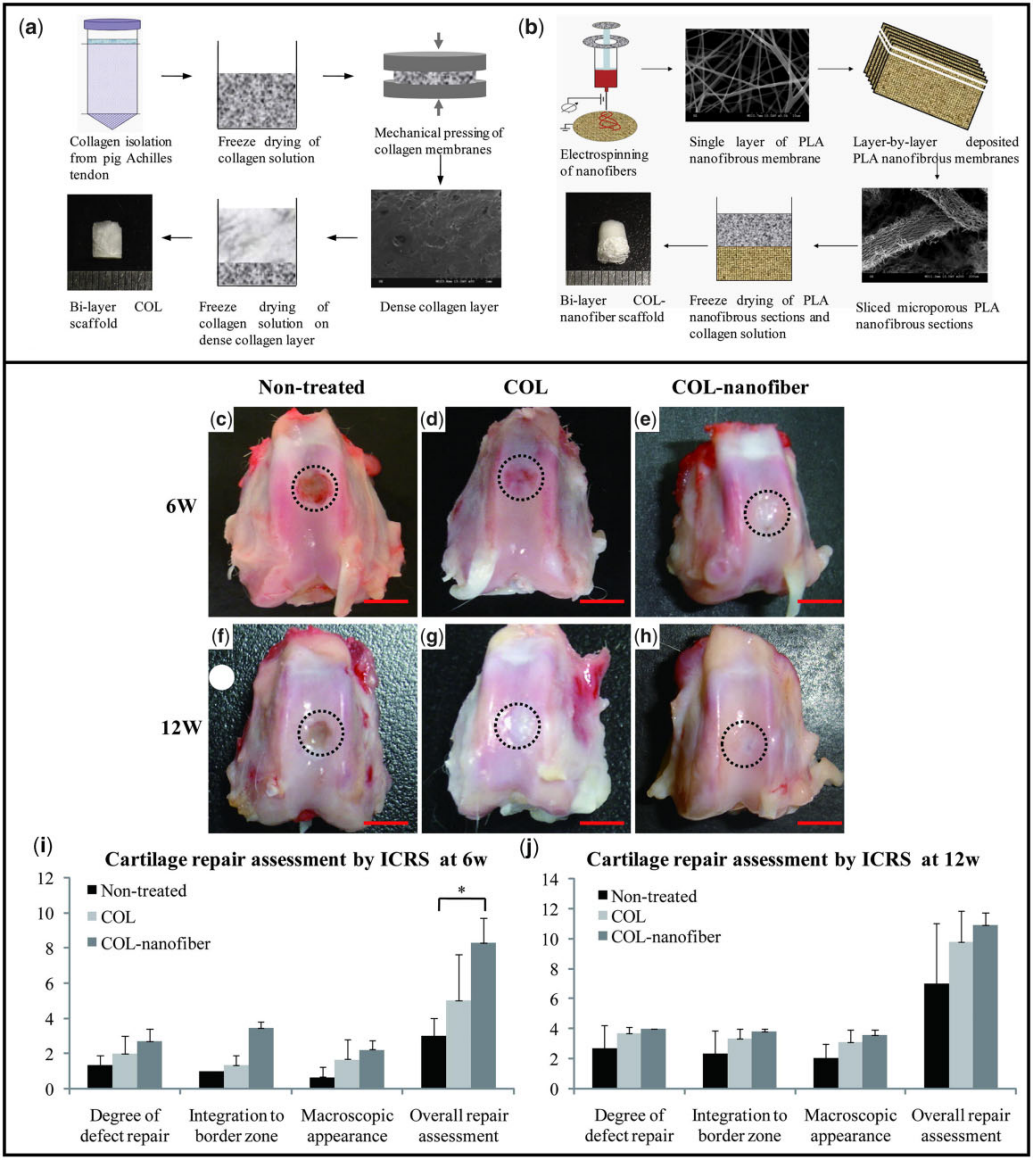
Collagen and electrospun PLA based biphasic scaffold in regeneration of osteochondral defects in rabbit model. (**a**) ‘All collagen’-based scaffold manufacturing method; (**b**) hybrid biphasic scaffolds fabrication of electrospun PLA and freeze-dried collagen; (**c**–**h**) gross morphology of the non-treated, all collagen (COL) and collagen-electrospun PLA fiber (COL-nanofiber) scaffolds; (**i**–**j**) ICRS scoring for the repair qualities (reprinted with permission from Elsevier).

Most often electrospinning is combined with other fabrication tools to achieve better hybridized properties compared to the individual techniques. Zhang *et al.* [130] fabricated a composite bi-layered scaffold integrating electrospinning and freeze-drying. PLA nanofibers were deposited layer-by-layer fashion to prepare the bone layer followed by the addition of collagen solutions on top of it to form an interface and the collagenous cartilage layer, followed by freeze drying (COL-nanofiber scaffold). hBMSCs were cultured and the COL-nanofiber scaffolds exhibited higher osteogenesis with almost 2-fold higher expression levels of osteocalcin (OCN) and RUNX2 compared to the control. After 12 weeks of transplantation in the rabbit femoral condyle *in vivo*, a combination of articular cartilage and fibrocartilage was observed in the COL-nanofiber groups, which may be attributed to the better overall osteochondral regeneration owing to a better quality of the subchondral bone repair. It is known that enhanced subchondral bone remodeling positively influences the regeneration of articular cartilage [131]. The enhanced chondrogenesis was also endorsed by the histological staining analysis (H&E and Safranin O) [130]. Figure 3 demonstrates the manufacturing schemes and the key results depicting the effectiveness of the electrospun nanofibrous phase in subchondral bone remodeling, in turn influencing better articular cartilage regeneration.

Liverani *et al*. [132] also developed a composite multi-layered scaffold integrating three techniques, sponge replication, freeze-drying and electrospinning. 45S5 Bioglass^®^ was used to fabricate the subchondral region, CS and alginate-based polymeric phase were chosen for the interfacial zone between the bioglass-based scaffold and the articular cartilage side. Finally, CS-based electrospun nanofibrous sheets were deposited as the superficial layer to mimic the cartilage phase. Other research groups also explored the beneficial aspects of electrospinning in osteochondral research as a customizable tool to deposit both bone and cartilage mimicking biomaterials with controlled spatial distribution and thickness [133–135]. Because of the nanofibrous nature, the sequentially deposited phases have ample scopes to interact or significantly overlap during the fabrication, facilitating a smoother interface generation in the osteochondral scaffolds.

#### AM of osteochondral tissue analogs

AM and bioprinting (a subclass of AM) is the concurrent workhorse in bio-manufacturing related research worldwide [136–138]. It is evident when the search string ‘osteochondral scaffold’ in google scholar (year sorted ‘since 2018 to present’) returns with more than 80% of scholarly articles, employing AM to fabricate anatomy/disease-specific complex scaffold systems for superior tissue integration and regeneration. Three-dimensional printing and bioprinting brought a paradigm shift in biofabrication where all classes of biomaterials (polymer, ceramics, metal, composites etc.) along with diverse cell types, protein, hormones, growth factors, small molecules etc. can be precisely deposited in a spatially controlled manner. Stereolithography (SLA), binderjetting, direct inkjetting, microextrusion (fused deposition modeling (FDM), bioplotting), selective laser sintering, electron beam melting and laser engineered net shaping, two-photon polymerization are the leading examples of advanced AM techniques. The best part of these next-generation manufacturing approaches is that they can be combined with other fabrication schemes (traditional or advanced) to achieve hybrid and/or superior properties. In this section, we will exert our endeavor to review the recent progress of AM in osteochondral regenerative engineering.

**Figure 4.**
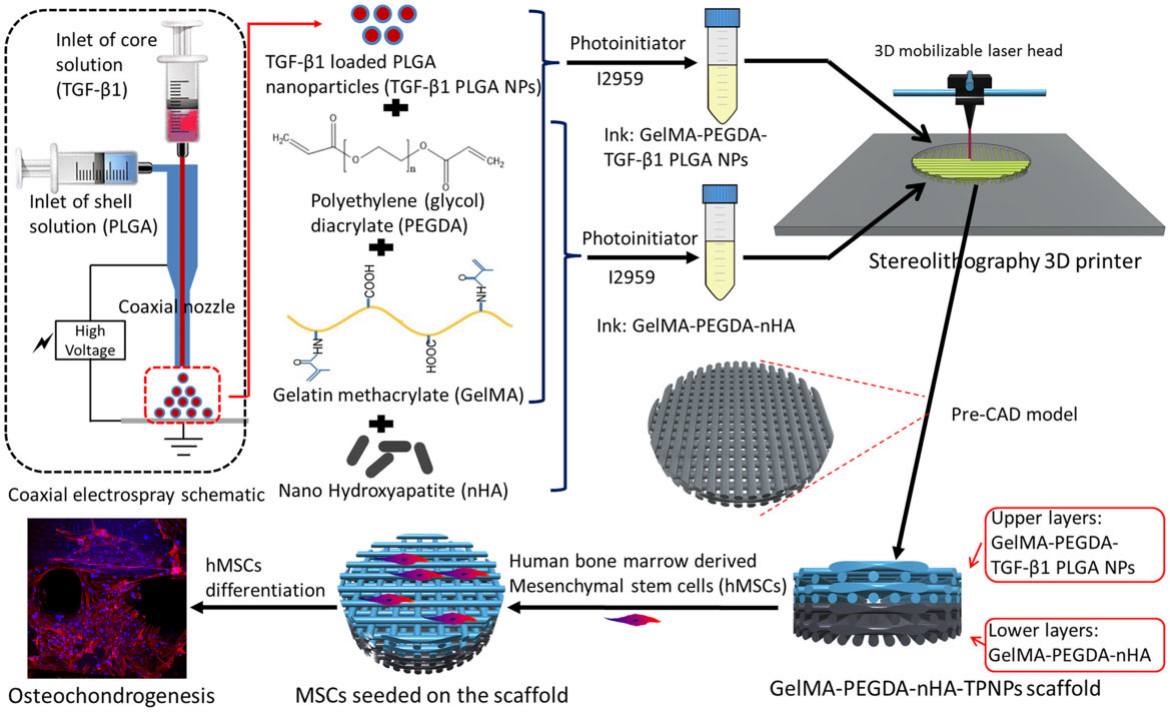
Schematic representation of the SLA-based 3D printing of osteochondral scaffolds using different nano-particles laden tissue specific bioinks. TPNPs, TGF-β1 laden PLGA nanoparticles (reprinted with permission from Elsevier).

SLA-based 3D printing was deployed to fabricate bi-layered osteochondral scaffolds using two different nano-particles laden bioinks dedicated to the cartilage and bone regions [139]. TGF-β1 laden PLGA nanoparticles were suspended in the combined hydrogel solution of gelatin methacrylate (GelMA) and poly-ethylene glycol diacrylate (PEGDA) as the cartilage bioink. Separately, nano-HAp was suspended in the same combined hydrogel medium of GelMA and PEGDA to be considered as the bone bioink. Figure 4 schematically represents the tissue-specific bioinks development and the printing process [139]. hMSCs were cultured in the 3D printed osteochondral scaffolds and the osteochondrogenesis were quantified using a set of histochemical staining (Alizarin red-S, Alcian Blue and Safranin O). After 2 and 4 weeks of culture, the expression levels of osteogenesis and chondrogenesis-related genes were analyzed using RT-PCR. The most intense histological staining was observed in the scaffolds with TGF-β1 encapsulated PLGA-based scaffolds (GelMA-PEGDA-nHAp/TGF-β1 PLGA NPs) implicating higher GAG deposition, which was consistent with the expression levels of the collagen II, SOX-9 and aggrecan. As expected, there was a significant improvement in the osteogenesis in the scaffolds having nano-HAp in the bone layer compared to the control [139].

**Figure 5.**
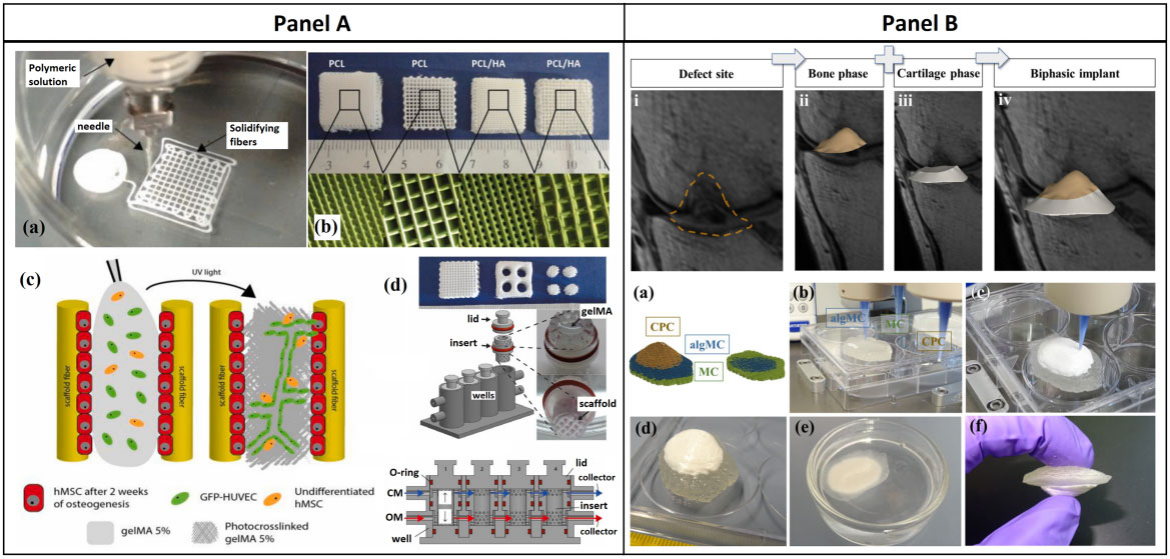
(**A**) PCL-based 3D printed bone scaffold along with GelMA layers as cartilage phase, *in situ* fabrication and maturation in dual-chamber bioreactor (reprinted with permission from Elsevier). (**B**) Fabrication of defect specific biphasic osteochondral architecture regenerated from patient MRI scan data of osteochondral lesion (source article published under open access, CC by license).

In a recent research, PCL and PCL/HAp-based osteochondral scaffolds were developed along with GelMA hydrogel having cartilage and vascularized bone phase using a specially designed AM approach [140]. The 3D printed osteochondral scaffolds were 3D cultured in a specially designed dual-chamber bioreactor (microphysiological system, MPS). In brief, PCL and PCL/HAp solution was printed using a microextrusion nozzle directly in a non-solvent to create the bone phase (Fig. 5a and b, A). Next, the bone scaffolds were kept in the lower half of the dual-chamber bioreactor, seeded with hMSCs and cultured in osteogenic differentiation media for 2 weeks. A mixture of hMSCs and HUVECs was suspended in GelMA and infiltrated the precultured bone scaffolds. The infiltrated gel was cured using UV radiation (for vascularization). After this, hMSCs laden GelMA was poured on the top of the osseous construct and photocured to obtain the cartilage phase. Two separate media flows in the MPS system were retained, although slight mixing at the interface was beneficial to generate the biomimicking calcified interface (Fig. 5c and d, A). The endothelial cells enhanced the efficacies of osteogenic differentiation of the hMSCs in the bioreactor system. The hMSCs laden gelMA region exhibited upregulation in the cartilage-related genes and intense staining of alcian blue implicated chondrogenesis and GAG deposition. This study emphasized the 3D culture in bioreactors to accelerate the regeneration of osteochondral tissues [140].

In the same year, another group developed a unique concept of defect-specific reconstruction of osteochondral lesions using advanced magnetic resonance imaging (MRI) data processing combined with micro-extrusion-based 3D bioplotting [141]. In brief, MRI scan data of a multi-zonal osteochondral defect in an *osteochondritis dissecans* (OCD) patient were obtained and the data were processed using a set of 3D image processing algorithms, followed by the generation of the 3D printable CAD file (*.stl) (Fig. 5i–iv, B). Three-dimensional printing strategy was developed by using multi-materials for different zones. A blend of alginic acid sodium salt and methylcellulose (algMC) was used as the extrudable cartilage phase, which after crosslinking with CaCl_2_ solution formed the articular-cartilage-like zone [141].

A sacrificial biomaterial methylcellulose (MC) was used as the supporting media for the overhanging structures during printing and calcium phosphate cement (CPC) paste was used as the bone phase (Fig. 5a–f, B). MRI scan is useful to capture the osteochondral defect geometry, because of the significant difference in density between bone and the cartilage, normal density attenuation-based X-ray CT is not capable to distinguish the cartilage tissue. On the other hand, osteochondral lesions and defects are patient-specific [141]. Taken together, it is imperative to realize that 3D printing and bioprinting are the current and next-generation fabrication tools in osteochondral regenerative engineering, having unparalleled efficacies in addressing heterogeneous and complex tissue regeneration in an anatomy/patient-specific manner. Table 2 summarizes the comprehensive landscape of AM in osteochondral regenerative engineering.

**Table 2.** Current progress in the design/defect-specific fabrication of osteochondral scaffolds using different AM approaches

AM methodology	Biomaterials and bioinks used	Cell lines used (co-printing or post-printing seeding)	Key results	Refs
SLA	1. GelMA + PEGDA + nHA as bone phase	hMSCs seeding after printing, *in vitro* differentiation	1. Intense histological staining in the scaffolds with TGF-β1 encapsulated PLGA and nano-HAp based scaffolds (GelMA—PEGDA—nHAp/TGF-β1 PLGA NPs)	[139]
	2. GelMA+PEGDA+TGF-β1 encapsulated PLGA as chondral phase		2. Higher expression levels of collagen II, SOX-9 and aggrecan in the cartilage layer	
Microextrusion	1. PCL/HAp as bone phase printing followed by hMSCs and HUVECs laden GelMA	hMSCs in both phases. Osteogenic and chondrogenic media flow in dual chamber bioreactor	1. *In vitro* vascularization	[140]
			2. Endothelial cells enhanced osteogenic differentiation of hMSCs	
	2. hMSCs laden GelMA as cartilage layer			
			3. A higher expression of collagen I and OPN as well as alizarin red staining	
			4. collagen II, SOX9 and aggrecan upregulation and intense staining of alcian blue in the chondral region	
3D plotting	1. Alginic acid sodium salt and methyl-cellulose (algMC) as cartilage phase	—	1. Defect specific multi-zonal reconstruction of osteochondral lesion using advanced MRI data from patients	[141]
	2. Calcium phosphate cement (CPC) as bone phase		2. High clinical implication: osteochondral lesions and defects are patient-specific and should be treated personalized manner	
FDM	1. 3D printed networks of PLA, PLGA and PCL fibers with (MSCs) laden alginate hydrogel as bone phase	MSCs, FPSCs and chondrocytes seeding during manufacturing.	1. Vascularised bone formation along with phenotypically stable cartilage formed on the surface, subcutaneously in mice model	[143]
	2. Same fibers with FPSCs and chondrocytes laden hydrogel on cartilage layer		2. Superior hyaline cartilage formation in caprine femoral condyle while compared with commercial control	
Microextrusion	Gradient porous PCL-PLGA fiber network. Higher porous cartilage side: CS modified PCL and lower porous bone side: β-TCP modified PLGA fibers	Post-printing culture with adipose-derived MSCs (ADMSCs)	1. Higher amount of sGAG in the cartilage layer of PCL/PLGA/CS scaffolds along with increased expression level of collagen II, SOX9 and aggrecan when compared to PCL/PLGA scaffolds.	[144]
			2. PCL/PLGA/βTCP bone region of the osteochondral scaffolds also exhibited enhanced ALP activity and mineralization when compared to the control counterpart	
SLA	1. 20% n-HAp as bone phase	hMSCs post-printing seeding *in vitro*	1. Highly interconnected porosity with nano-to-micro structure and spatiotemporal growth factor gradients	[145]
	2. 10% n-HAp as an intermediate phase		2. TGF-β1 encapsulated PLGA scaffold outperformed all control samples in GAG deposition	
	3. TGF-β1 encapsulated PLGA as cartilage phase		3. Osteochondral scaffold with nHA and TGF-β1 showed the highest mineral deposition in the bone phase	
Microextrusion	1. 15% GelMA hydrogel for cartilage zone	BMSCs seeding and *in vivo* implantation in rabbit osteochondral model	1. H&E staining shows a clear tidemark in the neo-tissue in the tri-layered scaffold group	[146]
	2. 20% GelMA and 3% n-HAp as intermediate zone		2. From gross-morphology, the tri-layered scaffold surpassed other groups in cartilage regeneration	
	3. 30% GelMA and 3% n-HAp as bone zone		3. The most intense orange color in the Safranin-O stained tri-layered scaffold demonstrates enhanced cartilage regeneration, *in vivo*	
Digital light processing (DLP)	Monophasic radially oriented osteochondral scaffold with GelMA, MSCs exosomes and cartilage-derived ECM	*In vivo* implantation in rabbit femoral condyle	1. Complete healing of cartilage from gross morphology analysis. Higher GAG deposition	[142]
			2. Enhanced subchondral ossification: higher ratio of bone volume to tissue volume (BV/TV) and trabecular thickness in the ECM/GelMA/exosome scaffolds	

Chen *et al*. [142] developed cartilage ECM-GelMA-exosome-based 3D printed osteochondral scaffolds with radially oriented channels using a desktop SLA-based 3D printer. The restorative efficacy of the MSC derived exosome loaded scaffolds in shielding the mitochondrial dysfunction and chondrocyte degeneration were evidenced *in vitro*. It was also found that the EVs (exosomes), loaded in the 3D printed scaffolds, could enhance chondrocyte migration as well as assisted in polarizing the synovial macrophage response toward an M2 phenotype. The 3D printed scaffolds were surgically implanted in the patellar groove of rabbit limbs, while the contralateral limbs were considered as untreated controls. Formation of hyaline like cartilage in the exosome containing scaffold groups was observed, followed by securing significantly higher ICRS score in the same group when compared with the untreated controls. Using a quantitative micro-CT analysis and HE staining approach, enhanced subchondral osteogenesis was also observed in the EVs laden scaffolds [142].

#### Scaffold-free organoid assemblage and microfluidics-based approaches

Since the inception of tissue engineering, biomaterials-based tissue-specific architectures/3D simulated organs engaged the regenerative research areas where the population of individual cells played a major role, irrespective of the fabrication methodologies. Nowadays organoid-based tissue engineering is nascent in the community where apart from single cells, miniature organs mimicking cellular aggregates, capable to perform specific biological functions like organs, are considered the building block for tissue-engineered architectures [147–149]. Most often, the tissue spheroids/organoids are prepared and cultured *in vitro*, before their use in tissue/regenerative engineering applications. With the recent advancements in developmental biology, it is known that *in vitro* simulation of biological processes during real skeletal development is potent to generate functional intermediate tissues which are capable to mature into full organs when implanted *in vivo* [150–152]. For example, to regenerate osteochondral cartilage in a biomimetic manner, the developmental pathway can be wisely followed where, the articulating long bones with articular cartilage terminals develop via a cartilage intermediate route, which is governed by mesenchymal condensation followed by chondrogenic maturation [153]. Induced pluripotent stem cells (iPSCs) have recently gained major interest in microtissue-spheroid-based cartilage tissue engineering given the unreliability of mesenchymal stem/stromal cells in regenerating hyaline cartilage, where in most cases fibrocartilage, rich in collagen I is formed. Albeit, autologous chondrocytes are the gold standard in repairing/regenerating hyaline/articular cartilage but the challenges in isolation and dedifferentiation hinder the large osteochondral defect treatment. The hyaline cartilage formed from iPSCs shows phenotypical stability after implantation in both rat and minipig osteochondral defect model [153, 154].

**Figure 6.**
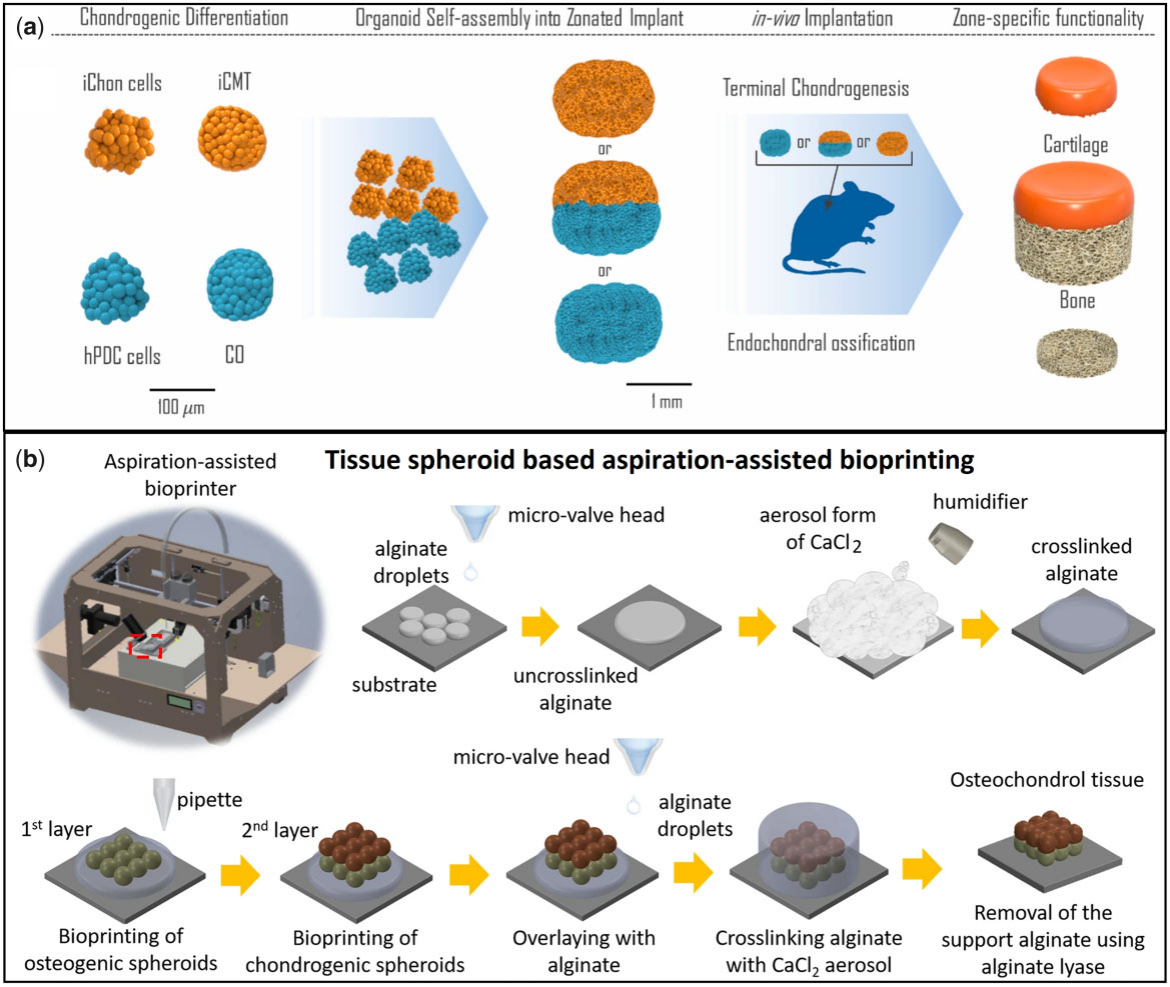
Organoid based biofabrication of osteochondral tissue complex using different manufacturing approaches. (**a**) iCMT and COs micro-tissue spheroids along with their corresponding parent cells iChon cells and hPDC cells were combined independently and fused zone-wise, *in vitro*, and subcutaneously implanted; (**b**) Aspiration-assisted bioprinting of tissue spheroids. Osteogenic spheroids were first layered followed by deposition of chondrogenic spheroids along with interfacial fusion (A: reprinted with permission from Elsevier; B: source article published open access, CC-by license).

Recently, Hall *et al*. [153] prepared iPSC-derived chondrocytes (iChon) along with bone-forming ‘callus organoids’ (COs) from human periosteum-derived cells (hPDCs) to develop chondral and osseous microspheroids. The hPDCs were cultured at different time points to achieve different intermediate tissues. For example, hPDSc micro-tissues cultured for 7 and 14 days represented the cartilaginous component while after 21 days, the microtissue system became ‘callus organoids (Cos)’ representing the hypertrophic part which would undergo endochondral ossification *in vivo*. Human induced pluripotent stem cells (hiPSCs) were cultured for 3 weeks in agarose-based micro-molds to form the induced cartilage microtissues (iCMT). Briefly, the mixture of ‘iChons’ and ‘iCMTs’ was used as the cartilage tissue precursor and the mixture of ‘hPDCs’ and ‘Cos’ was considered as the hypertrophied tissue precursor for endochondral ossification *in vivo*. The tissues were allowed to ‘fuse’ *in vitro* and implanted subcutaneously in immune-compromised mice. After 4 weeks, H&E and Safranin O/fast green staining showed two zones of bone and cartilage in the dual-layered construct. The mineralization was confirmed form human OCN immunostaining. The cortical bone along with bone marrow was also observed in the explanted COs zone of the dual-layered osteochondral construct. Figure 6a schematically represents the organoid-based assemblage of microtissue spheroids in developing bionic osteochondral graft [153].

In another recent study, a comprehensive work-flow to bioprint ADSC-derived chondrogenic and osteogenic tissue spheroids layer-by-layer was reported to construct osteochondral tissue complex [155]. After the bioprinting, the zonal interfaces fused and the tissue phenotypes in both regions remained unaltered. In brief, ADSCs spheroids were prepared for 5 days followed by the addition of chondrogenic and osteogenic differentiation media in selected well plates. The spheroids were allowed to differentiate for another 21 days. The osteogenic spheroids were bioprinted first, followed by the chondrogenic spheroids which were directly deposited on the last layer of the osseous phase and the entire bi-phasic construct remained sheltered in the alginate matrix, crosslinked using CaCl_2_ aerosol spray. The construct was cultured in co-differentiation media for 1 week to ensure the complete fusion of the interfacial region of the chondro- and osseous phases, followed by citrate-mediated dissolution of the protective alginate phase. H&E staining showed the compactness of the osteochondral tissue complex having a distinguishable interfacial region. Alizarin red and toluidine blue staining assay demonstrated GAG formation throughout the cartilage phase and mineral deposition in the osseous region [155]. The ‘scaffold-free’ method of complex tissue bioprinting broadened the scopes of clinical research in autologous stem cells-based patient-specific osteochondral regenerative treatments (Fig. 6b).

Since the last decade, organ-on-a-chip based research expanded significantly in drug screening, modeling several diseases comprising complex tissues and interfaces [156–158]. It is not always feasible to involve animal models in first-generation experiments where the concepts and methodologies are still evolving [159]. Hence, it is necessary to have a simulated physical platform where different biofluid flow along with the influences of multiple tissues can be investigated. Microfluidics-based organ-on-a-chip models facilitate such customized *in vivo* mimicking platforms. The individual and synergistic effects of surface area, biomaterial-based matrix properties, growth factors, hormones, small biomolecules and novel drugs are investigated on different cellular and tissue behaviors. Against this backdrop, several attempts to model osteochondral tissue regeneration in different hydrogel matrices along with OA disease modeling and OA drug screening are reported [159–161].

Lin *et al*. [160] developed a proof-of-concept of osteochondral tissue on chip, employing modified iPSCs cells. iPSCs were further induced in ‘induced mesenchymal progenitor cells (iMPCs)’ followed by encapsulation in photo-crosslinked gelatin hydrogel and 3D cultured in a dual flow chip system. The bottom part of the cell encapsulated hydrogel was exposed to the flow of osteogenic media whereas the top part directly interacted with chondrogenic media. After 28 days of *in vitro* simulated culture, the generation of cartilage and bone tissues were confirmed from histology and PCR-based osteogenic and chondrogenic gene expression analyses. Alcian blue and alizarin red staining confirmed the cartilage and bone formation in the bottom and top components of the hydrogel construct. Gene-expression studies also confirmed the multi-fold increment in the aggrecan and collagen II expression level on the top cartilage part compared to the bottom bony region, while the bone marker protein expressions (OCN and BSP) showcased the counter trend as expected [160].

## Off-the-shelf osteochondral scaffolds: recommendations in a translational perspective

In laboratory-scale investigations, it is common to combine live cells, growth factors, biomolecules etc. to enhance the regenerative efficiency of osteochondral scaffolds. However, in translational aspects, it is difficult to consider the scaffolds having live cells, growth factors proteins etc., due to strict federal regulations on cellular and/or biomolecule-loaded scaffolds where they are considered ‘high-risk devices’ [162–164]. In some cases, surgeons collect the biopsy of the patient containing autologous chondrocytes and send it to approved companies. The autologous cells are cultured and grown on a porcine collagen matrix and shipped to the surgeon back for the implantation in defect site (two-step procedure). This is not always straight forward and affordable therapeutic approach. Now it is apparent why most of the commercial scaffolds [Chondromimetic (Tigenix NV), MaioRegen (Finceramica), TruFit (Smith & Nephew), Biomatrix CRD, MACI (Vericel), Chondro3 (Locate-Bio), MaioRegen, Agili-C etc.] are off-the-shelf, acellular and not modified with any protein or growth factors. They often fail to compete with the cellular scaffolds investigated in a laboratory environment.

A preclinical study is the first step for the translation of a novel osteochondral scaffold toward market [108, 165, 166]. In this context, we will use market and clinic in an overlapping manner as both are interdependent. At first, the biocompatibility of the novel scaffold should be assessed subcutaneously in a mouse or rat model. On the achievement of satisfactory outcomes through tissue inspection (histology, immunostaining etc.), one should plan the proof-of-principle study in rabbit or similar animal models. In all animal trials, institutional and/or federal animal study-related ethical committee/board approval is a must. After the successful establishment of the proof of principle results, pilot studies in small ruminants (goats, sheep etc.), dogs, pigs or horses should be designed conforming with the ‘3R’ principle (replacement, reduction and refinement) [167–169]. In most of the practices, the osteochondral scaffolds are implanted in the condyle or trochlear sites of the knee joints of the hind limbs. For example, for osteochondral scaffolds for implantation in the rabbit model, the scaffold diameter should not exceed 3.5 mm with 3 mm height. Whereas in the minipig model, the diameter should be in the range of 5–8 mm along with the height of 7–10 mm, which should be decided based on the available cartilage and subchondral bone thickness [170–172]. The total period and the intermediate study intervals of the preclinical studies are important to record the dynamic progress of osteochondral healing and regeneration. While the proof-of-principle studies in small animals should be followed up for 8–12 weeks, 6–12 months follow-up studies are generally preferred for the pilot studies in larger animals. As the preclinical studies should endeavor to mimic the clinical procedures as closely as possible, animal age, cartilage maturity and thickness, calcified cartilage and subchondral bone plate anatomy should be considered in the study design [166, 173]. Others (Mohan *et al*., ‘Gradient osteochondral scaffold system’ section) explored the effectiveness of the osteochondral scaffold-based therapeutic approach in larger-size animals while comparing the results with the clinically practiced procedures. For example, critical-sized gradient osteochondral scaffolds were used in skeletally mature sheep models for long-term regeneration where microfracture, a ‘clinical standard of care’ was used as the control. The surgical operative procedure (unilateral or bilateral) and the defect model (acute or chronic) should also be considered to achieve the suitable performance of the scaffold system in the clinical environment. Once the results are satisfactory through histological and immunohistochemical staining, micro-CT, gross morphology and cartilage repair scoring, biodegradation and release kinetics, mechanical properties etc., the next step is to proceed for the Food and Drug Administration (FDA) approval/clearance for commercialization.

All cartilage and osteochondral devices are Class III devices (‘high risk’, implantable device) irrespective of their cellular or acellular nature [174]. Apart from osteochondral scaffolds, this also covers collagenous matrices, HA injection etc. Class II devices are mostly non-invasive or can be used around the peripherals of the body. If a device is Class II categorized, a clinical trial may not be required and in this case, a post-review 510(k) ‘clearance’ can be issued by the FDA to bring the device to market. As there is no Class II device capable to treat articular cartilage and osteochondral defect, 510(k) is not straightforward for the osteochondral device. As the category of cartilage/osteochondral regenerative devices falls under Class III device (FDA product code—‘NCO’), before commercialization a Premarket Approval (PMA) by the FDA would be necessary. To obtain a PMA, the osteochondral device has to go through a systematic clinical trial involving human subjects. If the *in vitro* and preclinical trial performances are convincing after review, FDA may permit the clinical trial, known as Investigational Device Exemption (IDE). The detailed guidelines for the human clinical trial of osteochondral implantable devices are beyond the scope of this article and can be found elsewhere [174–177].

At any developmental stage between the *in vitro* and proof-of-principle studies, a patent can be filed if the features of the osteochondral scaffolds are novel and not reported in the prior art. Institutional intellectual property departments or independent patent lawyers can be consulted to submit the disclosure to the federal office for the grant. During this process, the technology readiness level (TRL, Level 1–9) and manufacturing readiness level (MRL, Level 1–10) should be critically evaluated [178, 179]. Although strict adherence to all the technology readiness levels (up to 9) is mandatory for the strategic sectors (defense, aerospace etc.), achievement up to TRL 6 is sufficient in biomaterials and biomedical engineering as a consensus, to initiate the commercialization procedures (large animal trial, IDE approval, PMA application) [178]. Good Laboratory Practice (GLP) is another important aspect to consider during the preclinical trial. Whether during the proof-of-principle studies GLP is not strictly required by the FDA, in the pivotal studies in larger animals, it is recommended to follow GLP guidelines for a satisfactory review outcome for the approval of IDE [180]. After the PMA is approved and the technology is commercialized by industry or collaborative start-up, Good Manufacturing Practice (GMP) is an essential aspect to be considered for all biomedical devices. FDA published the GMP guidelines (21 CFR Part 820) which are very similar to the ISO 13485 depicting the EU GMP in the Europe [181]. At this stage, the device business model such as B2B (business to business), B2C (business to consumer) along with the recent conversion toward B2H (business to human) should be structured.

Finally, we return to our initial argument to reiterate that, the successful off-the-shelf commercial osteochondral scaffolds are acellular and their intrinsic biocompatibility and the efficacy of host tissue integration should be at the highest level. The major challenge is to select the most suitable osseous and chondrogenic biomaterial combination, having the bone and cartilage mimicking gradient microstructure (porosity volume and shape distribution for example) and mechanical properties. An uncompromised adherence to these attributes will enable the acellular and unmodified devices to compete very closely with the live cells, growth factor and biomolecules-loaded osteochondral scaffolds, mostly investigated for research and development purposes. The inherent challenges, comprehensive state-of-the-art and systematic recommendations outlined in this article should be able to guide the early career researchers to promote osteochondral scaffolds/implants/devices in the healthcare market. This will potentially address the availability and affordability of osteochondral scaffolds/implants to the economically challenged and elderly population in the country and beyond.

## Conclusion

In this review, a significant endeavor has been made to highlight the key challenges in osteochondral regenerative engineering. There are numerous structural as well as functional inhomogeneities in the tissue interface and inherent difficulties in the bionic regeneration/modeling of the underlying transitions in the tissue complex. Global attempts have been reported where the researchers successfully designed and demonstrated novel scaffold systems in the efficacious regeneration of hyaline cartilage and subchondral bone phases. The hierarchical zones in both the cartilage and subchondral bones have been modeled using suitable ceramic, polymer and hydrogel-based biomaterials along with various growth factors, EVs, biomolecules etc. A wide range of biofabrication approaches ranging from conventional, advanced and/or hybrid routes was discussed comprehensively along with the key results, to portray the state-of-the-art of osteochondral regenerative engineering. Despite the decades-long endeavors to address this crucial healthcare segment, there exist outstanding ‘death valleys’ that are yet to be bridged. One such lacuna is the poor performance of the commercially available acellular, unmodified osteochondral scaffolds in comparison with the live cells, growth factors laden scaffolds, which cannot be used ‘on demand’ due to regulatory restrictions. Development and the federal approval of more biocompatible matrices along with biomimicking microstructures are the calls of the hour. These new generation bionic scaffolds should be able to regenerate/repair osteochondral lesions/defects, even without the use of live cells and growth proteins. To this end, significant efforts are invested in this review to provide a concise but comprehensive recommendation set of various regulatory aspects, intermediate procedures along with their interrelations and overall progressive stages. This endeavor should promote the current laboratory-scale developments of osteochondral scaffolds toward more advanced ‘bench to bedside’ and/or ‘bedside to bench to bedside’-based research.

## Supplementary Material

rbac109_Supplementary_Data
