# Supplementary material for: Osteochondral regenerative engineering: challenges, state-of-the-art and translational perspectives
Source: Regen Biomater. 2022 Dec 26;10:rbac109. doi: 10.1093/rb/rbac109 (PMC9845524; doi:10.1093/rb/rbac109)
Supplement: rbac109_Supplementary_Data [file rbac109_supplementary_data.docx]

**Authors’ responses against Reviewer’s comments on the Manuscript (RB-2022-228): Osteochondral Regenerative Engineering: Challenges, State-of-the-Art and Translational Perspectives**

The authors are grateful to the Reviewers and the Editor for the constructive comments and suggestions. The authors have thoughtfully analyzed and addressed all the comments in the revised version. Please find the authors’ responses below:

**Reviewer: 1**

Therapeutic extracellular vesicles (EVs) are an emerging area in regenerative medicine. There should be a section on EVs, particularly EVs derived from mesenchymal stromal cells (MSCs) that have been reported to mediate the therapeutic efficacy of MSCs in tissue repair including bone and cartilage repair.

Several studies have reported the incorporation of EVs into scaffolds for repair of osteochondral defects. Some considerations on the design, fabrication, and modification of the scaffolds for loading of the EVs should be discussed, and how these could impact the sustained release, integrity, bioactivity of the EVs.

**Author Response:** The authors appreciate the constructive comments of the reviewer in improving the quality of this manuscript further. An introductory segment is incorporated in the section 2.3 with section heading of “Challenges in biomaterials, biomolecules and growth factors selection”. The introduced text is quoted: “Similarly, extracellular vesicles (EVs) released by mesenchymal stem/stromal cells, recently grabbed the attention in view of their regulatory capabilities in targeted bone and cartilage regeneration. EVs mediate cell signalling, influences major biological activities such as cell growth, migration and proliferation. Briefly, the bi-lipid layered EVs breakdown in the extracellular space and the cargo is instantly released to interact with the target cells. Over the last 5 years, EVs are being investigated for their therapeutic efficacies in osseous and chondral regeneration” (manuscript page number 7).

Further, a comprehensive case study is included where osteochondral scaffolds were 3D printed using hydrogel laden exosomes (a variant of EVs) and their efficacies in osteochondral regeneration were quantified in vitro and in vivo (rabbit model). Please refer to the section 3.3.2 with the section heading of “Additive manufacturing of osteochondral tissue analogs”. The newly incorporated text is quoted: “Chen et al developed cartilage ECM-GelMA-exosome-based 3D printed osteochondral scaffolds with radially oriented channels using a desktop stereolithography based 3D printer. The restorative efficacy of the MSC derived exosome loaded scaffolds in shielding the mitochondrial dysfunction and chondrocyte degeneration were evidenced in vitro. It was also found that the EVs (exosomes), loaded in the 3D printed scaffolds, could enhance chondrocyte migration as well as assisted in polarizing the synovial macrophage response toward an M2 phenotype. The 3D printed scaffolds were surgically implanted in the patellar groove of rabbit limbs, while the contralateral limbs were considered as untreated controls. Formation of hyaline like cartilage in the exosome containing scaffold groups was observed, followed by securing significantly higher ICRS score in the same group when compared with the untreated controls. Using a quantitative micro-CT analysis and HE staining approach, enhanced subchondral osteogenesis was also observed in the EVs laden scaffolds” (manuscript page number 22).

**Reviewer: 2**

This is a valuable review paper that comprehensively summarized the challenges in osteochondral regeneration from aspects of biomaterials, sourcing cells, biomolecules and growth factors, as well as approaches for design and fabrication of scaffolds. It highlighted the significance of biomimicking the gradients in porous microstructure, cells and ECM orientation, especially for inducing vascularization in the subchondral bone region and avascular nature in the adjacent cartilage layer. New biofabrication tools, such as additive manufacturing, electrospinning, and microfluidics techniques in developing biomimetic osteochondral scaffolds were intensively introduced. In addition, the challenges in translational researches were emphasized, aiming at the biomaterial-based acellular scaffold systems with uncompromised biocompatibility and osteochondral regenerative capability. This review could be a suggestive guidance and useful reference for researchers working in tissue engineering area. I would recommend to accept it for publication with only one mirror concern in section 2.2, where the authors referred autogenous chondrocyte implantation as the “gold standard”. Although ACI (or MACI) had developed for years, and had been approved by FDA in 2016, it was not considered as “gold standard” since the difficulty in harvest and the dedifferentiation during the expansion in vitro.

**Author Response:** The authors express gratitude and sincerely thank the reviewer for the appreciation of the manuscript. We have removed the terminology “gold standard” and updated it in the manuscript (section 2.2, manuscript page number 4).

We hope that we could address both the reviewer’s comments satisfactorily.
